# Bacteriological Quality and Biotoxin Profile of Ready-to-Eat Foods Vended in Lagos, Nigeria

**DOI:** 10.3390/foods12061224

**Published:** 2023-03-13

**Authors:** Oluwadamilola M. Makinde, Michael Sulyok, Rasheed A. Adeleke, Rudolf Krska, Chibundu N. Ezekiel

**Affiliations:** 1 Department of Microbiology, Babcock University, Ilishan Remo 121103, Ogun State, Nigeria; 2 Unit for Environmental Sciences and Management, North-West University (Potchefstroom Campus), Potchefstroom 2531, South Africa; 3 Department of Biological Sciences and Biotechnology, Caleb University, Lagos 106102, Nigeria; 4 Department of Agrobiotechnology IFA-Tulln, Institute of Bioanalytics and Agro-Metabolomics, University of Natural Resources and Life Sciences, Vienna, Konrad-Lorenz-Strasse 20, 3430 Tulln an der Donau, Austria; 5 Institute for Global Food Security, School of Biological Sciences, Queen’s University Belfast, University Road, 19 Chlorine Gardens, Belfast BT9 5DL, Northern Ireland, UK

**Keywords:** *aadun*, aflatoxin, *eko*, foodborne bacteria, food contaminants, food safety, fumonisin, *kokoro*, plant toxins, phytoestrogenic phenols

## Abstract

A comprehensive study of bacterial and biotoxin contaminants of ready-to-eat (RTE) foods in Nigeria is yet to be reported. Hence, this study applied 16S rRNA gene sequencing and a dilute-and-shoot LC-MS/MS method to profile bacteria and biotoxins, respectively, in 199 RTE food samples comprising *eko* (n = 30)*,* bread (n = 30), shawarma (n = 35), *aadun* (n = 35), biscuits (n = 34), and *kokoro* (n = 35). A total of 631 bacterial isolates, clustered into seven operational taxonomic units, namely *Acinetobacter*, *Bacillus*, *Klebsiella*, *Proteus* and *Kosakonia*, *Kurthia*, and *Yokenella*, that are reported for the first time were recovered from the foods. One hundred and eleven metabolites comprising mycotoxins and other fungal metabolites, phytoestrogenic phenols, phytotoxins, and bacterial metabolites were detected in the foods. Aflatoxins, fumonisins, and ochratoxins contaminated only the artisanal foods (*aadun*, *eko,* and *kokoro*), while deoxynivalenol and zearalenone were found in industrially-processed foods (biscuit, bread, and shawarma), and citrinin was present in all foods except *eko*. Mean aflatoxin (39.0 µg/kg) in artisanal foods exceeded the 10 µg/kg regulatory limit adopted in Nigeria by threefold. Routine surveillance, especially at the informal markets; food hygiene and safety education to food processors and handlers; and sourcing of high-quality raw materials are proposed to enhance RTE food quality and safeguard consumer health.

## 1. Introduction

**Figure 1:**
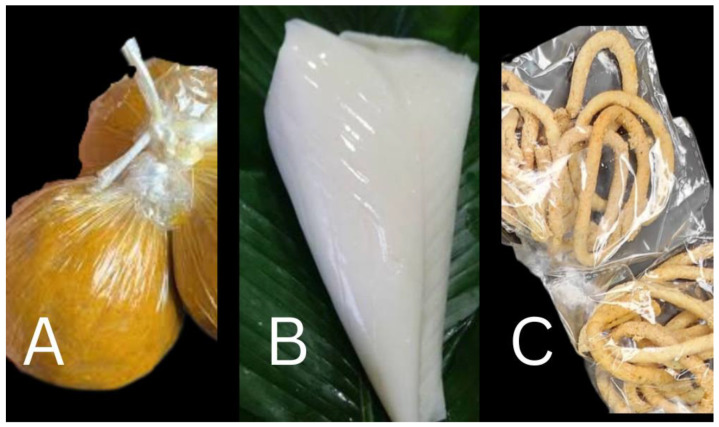
A pictorial representation of artisanal produced ready-to-eat foods. (**A**) *Aadun*, (**B**) *eko*, (**C**) *kokoro*.

Food products vended and consumed without any form of additional processing or preparation are commonly termed ready-to-eat (RTE) foods [1,2,3]. RTE foods, which are widely consumed globally, can be classified into artisanal and industrial foods based on the mode of production. In Nigeria, *aadun* (roasted mix of cassava flour and maize flour), *eko* (solidified form of cooked fermented maize gruel)*,* and *kokoro* (fried mix of maize flour and groundnut flour) (Figure 1) are produced through artisanal methods, whereas biscuits, bread, and shawarma, all made from wheat flour, are made industrially [2,3,4,5,6]. The aforementioned foods are among the commonest RTE foods consumed in Nigeria, with those produced through the artisanal methods being more common in the southwestern region to which Lagos State belongs. RTE foods are appealing and widely accepted by consumers due to their availability, especially for individuals in transit, and their relatively cheaper costs compared to most exotic foods [3,7,8]. These RTE foods are consumed by individuals of different age groups (children, adolescents, and adults). However, RTE foods are prone to contaminants (e.g., foodborne bacteria and biotoxins) that access the food as a result of poor handling of raw materials from the farm through storage, low personal hygiene during production and preparation of the foods, and substandard practices during packaging and sale of the finished products [3,9,10,11].

Foodborne bacteria such as *Aeromonas* spp., *Bacillus cereus*, *Enterococcus* spp., *Escherichia coli*, *Klebsiella pneumoniae*, *Listeria monocytogenes*, *Proteus vulgaris*, *Pseudomonas aeruginosa*, *Salmonella* spp., and *Shigella* spp. have been reported to contaminate RTE foods, using them as vehicles of entry into the human body [1,2,12,13]. The outcomes of exposure to these foodborne bacteria include a range of foodborne illnesses such as gastroenteritis, diarrhoea, typhoid fever, and dysentery. On the other hand, biotoxins comprise a range of toxins of microbial (e.g., bacterial and fungal toxins), animal, and plant origins [14]. Mycotoxins, which are toxic secondary metabolites of fungal origin that contaminate foods, are the most important biotoxins of food safety and public health relevance [15,16]. Several studies have reported the presence of diverse mycotoxins such as aflatoxins, fumonisins, ochratoxins, trichothecenes (deoxynivalenol (DON) and nivalenol (NIV)), zearalenone (ZEN), and the emerging toxins (alternariol (AOH), beauvericin (BEA), enniatins, moniliformin (MON), and tenuazonic acid) in RTE foods [17,18,19,20,21,22,23]. Exposure to these dietary toxins could result in cancers, suppression of the immune system, and acute or chronic organ toxicities in humans and animals [24].

Despite the available empirical data on foodborne bacterial or mycotoxin contamination of RTEs globally, there is heavy dependence on poorly processed and packaged RTE foods in countries (e.g., Nigeria) categorized as low and middle income [3]. To the best of our knowledge, there is no report on the combined surveillance data of bacterial contaminants and mycotoxins in RTE foods in Nigeria. Such data are necessary to elucidate the broad picture of food safety contaminants in these highly sourced foods. Moreover, the World Health Organisation of the United Nations reports a foodborne disease burden of 40% among children [25], who are also major consumers of the RTE foods. Consequently, this study aimed to ascertain the bacteriological quality and biotoxin profile of RTE foods vended in Lagos State, Nigeria, with a view of promoting consumer health.

## 2. Materials and Methods

### 2.1. Food Samples

One hundred and ninety-nine RTE food samples comprising six RTE food types, namely *aadun* (n = 35), biscuits (n = 34), bread (n = 30), *eko* (n = 30), *kokoro* (n = 35), and shawarma (n = 35), were purchased from vendors in three purposely selected major markets in Lagos State, Nigeria, between June and September 2019. The markets were situated at Agege (6.6198° N, 3.3249° E), Iyana-Ipaja (6.6106° N, 3.2958° E), and Ikorodu (6.6169° N, 3.5081° E) and were selected for the high patronage of RTE foods. In each market, a minimum of 10 samples of each RTE food type was collected such that the total samples per market were 65, 65, and 69 for Agege, Iyana-Ipaja, and Ikorodu, respectively. Each food sample (approximately 300 g) consisted of at least three sub-samples randomly pooled from the vendor’s tray. Food samples were purchased as packaged by vendors, immediately placed in an ice-box maintained at 4 °C, and transported to the laboratory for analysis within 24 h.

### 2.2. Bacteriological Analysis of Food Samples

Twenty grams of each food sample was aseptically taken into a sterile mortar, mashed, and homogenized in 200 mL of peptone water. The homogenized samples were serially diluted by tenfold, and 1 mL aliquots were spread plated on the general-purpose nutrient agar (Oxoid, Basingstoke, UK) and selective MacConkey agar (Oxoid, Basingstoke, UK) plates. Duplicate plates for each dilution were spread plated per food sample and incubated at 37 °C for 24 h. Total bacterial count (TBC) and total enterobacterial count (TEC) were enumerated. Thereafter, an assessment of the cultural characteristics (colony colour, elevation, and edges) and examination of cell morphology (colour, shapes, and arrangement) after Gram staining were performed for each isolate. Representative isolates selected after the examinations aforementioned were maintained as pure cultures on nutrient broth fortified with 2% glycerol at −20 °C.

### 2.3. Genomic DNA Analysis of Foodborne Isolates

#### 2.3.1. Extraction of Genomic DNA and PCR

Genomic DNA of overnight bacterial cultures in nutrient broth were extracted using the ZR Fungal/Bacterial DNA MiniPrep kit (Zymo Research, Carlifonia USA) according to instructions of the kit manufacturer. Universal primers 27F (5′-AGAGTTTGATCCTGGCTCAG-3′) and 1492R (5′-TGACTGACTGAGGCTACCTTGCGA-3′) [26] were employed in the amplification of the 16S rRNA gene of the isolates. The PCR reaction contained 12 μL of a double-strength DreamTaq PCR Master Mix (Thermo Fischer Scientific, Waltham, MA, USA), 1 μL template DNA, 1 μL each of forward and reverse primers (10 mM), as well as nuclease-free water to a final reaction volume of 25 µL. The thermo-cycling (Vacutec Apha cycler 1 PCRMax, Roosevelt Park, South Africa) conditions consisted of an initial denaturation step at 95 °C for 5 min, 35 denaturation cycles at 94 °C for 30 s, an annealing step at 61 °C for 30 s, an extension step at 72 °C for 5 min, and a final extension step at 72 °C for 7 min. Amplicon sizes (bp) were verified using 1% agarose gel electrophoresis.

#### 2.3.2. 16S rRNA Gene Sequencing and Phylogenetic Analyses

Purified amplicons were sequenced at Inqaba Biotechnical Industries (Pty) Ltd., Pretoria, South Africa. The PRISM™ Ready Reaction Dye Terminator Cycle Sequencing Kit (Sanger sequencing) with reverse primer 907R (5′-CCGTCAATTCCTTTGAGTTT-3′) was employed in the sequencing PCR reactions; thereafter, the products were electrophoresed with a model ABI PRISM^®^ 3500XL DNA Sequencer (Applied Biosystems, Foster City, CA, USA). The high-quality partial 16S rRNA gene sequences obtained from the isolates were clustered into operational taxonomic units (OTUs) at 99% similarity using the Mothur software version 1.37.2 [27]. Thereafter, taxonomies were assigned to OTU representative sequences by aligning the sequences against the *EzBiocloud* database (http://www.ezbiocloud.net/ accessed on 12 December 2020) [28]. MEGA version X [29] was used to phylogenetically reconstruct the OTU representative sequences with closely related GenBank sequences as previously described by Adedeji et al. [30]. All the sequences obtained in this study were deposited at NCBI GenBank under the accession numbers MW448721, MW448743, MW449471–MW449476, MW465227–MW465230, and MW473709–MW473713.

### 2.4. Determination of Amylase and Haemolysin Production in Foodborne Isolates

The potential of the recovered isolates to spoil foods through starch hydrolysis and cause foodborne illnesses were determined by assaying for production of amylase and haemolysin, respectively. These tests, though not definitive, are easy to perform in a resource-scarce setting. Amylase and haemolysis tests were conducted on each isolate using freshly prepared starch agar (0.5% NaCl, 0.5% peptone, 0.3% yeast extract, 1.5% Bacto agar, and 0.2% soluble starch, *w*/*v*) and blood agar (5% antibiotics-free human blood in 1000 mL nutrient agar, *v*/*v*), respectively [31].

### 2.5. Multi-Mycotoxin Analysis of RTE Food Samples

Biotoxins in food samples were profiled according to a dilute and shoot LC-MS/MS method [32]. The biotoxins determined by the method included approx. 800 metabolites comprising bacterial, fungal, and plant toxins.

#### 2.5.1. Chemicals

Methanol (LC gradient grade) and glacial acetic acid (p.a) were purchased from Merck (Darmstadt, Germany), acetonitrile (LC gradient grade) from VWR (Leuven, Belgium), and ammonium acetate (MS grade) from Sigma-Aldrich (Vienna, Austria). Mycotoxin standards were donated by various research groups or purchased from various commercial sources. Water was purified successively by reverse osmosis using an Elga Purelab ultra-analytic system from Veolia Water (Bucks, UK).

#### 2.5.2. Extraction of Metabolites and Estimation of Apparent Recoveries

Next, 5 g of the food sample was weighed into a 50 mL polypropylene tube (Sarstedt, Nümbrecht, Germany) and homogenized with 20 mL of extraction solvent (acetonitrile/water/acetic acid 79:20:1, *v*/*v*/*v*). Thereafter, samples were extracted for 90 min on a GFL 3017 rotary shaker (GFL, Burgwedel, Germany) and then diluted with 20 mL of the extraction solvent. Due to sufficient sedimentation by gravity of the diluted extracts, a direct injection of the diluted extract into the LC-MS/MS instrument was performed. To determine apparent recoveries of the analytes, 0.25 g of five samples were spiked. The solvents in the spiked samples were allowed to evaporate overnight at ambient temperature such that an equilibrium was established between the analytes and samples. Spiked samples were then extracted (in 1 mL solvent), diluted, and analysed as described earlier [32]. The accuracy of the method was verified by participation in inter-laboratory comparison studies organized by BIPEA (Gennevilliers, France).

#### 2.5.3. LC-MS/MS Parameters

LC-MS/MS screening of the biotoxins was performed with a QTrap 5500 LC-MS/MS System (Applied Biosystem, Foster City, CA, USA) equipped with a TurboIonSpray electrospray ionisation (ESI) source and a 1290 Series HPLC System (Agilent, Waldbronn, Germany). Chromatographic separation was performed at 25 °C on a Gemini^®^ C18-column, 150 × 4.6 mm i.d., 5 μm particle size, equipped with a C18 4 × 3 mm i.d. security guard cartridge (Phenomenex, Torrance, CA, USA). The chromatographic method and chromatographic and mass spectrometric parameters are as described by [32] Sulyok et al. (2020). ESI MS/MS was performed in the time-scheduled multiple reaction monitoring (MRM) mode both in positive and negative polarities in two separate chromatographic runs per sample by scanning two fragmentation reactions per analyte. The MRM detection window of each analyte was set to its expected retention time of ±20 s and ±26 s in the positive and the negative modes, respectively. Confirmation of positive analyte identification was obtained by the acquisition of two MRMs per analyte (with the exception of moniliformin (MON) and 3-nitropropionic acid (3-NPA), which exhibited only one fragment ion). This yielded 4.0 identification points according to Commission Implementing Regulation (EU) decision 2021/808 [33]. The LC retention time and the intensity ratio of the two MRM transitions also agreed with the related values of an authentic standard within 0.03 min and 30%, respectively. The limits of detection (LOD) and quantification (LOQ) ranged from 0.01–25.0 µg/kg and 0.03–75.0 µg/kg, respectively (Supplementary Table S1).

### 2.6. Data Analysis

Data on occurrence of bacteria and biotoxins in the RTE foods were analysed using SPSS Statistics package version 20.0 (SPSS Inc., Chicago, IL, USA). Descriptive statistics were applied to elucidate the distribution of bacteria and mycotoxin levels in the RTE food types.

## 3. Results and Discussion

### 3.1. Distribution of Bacteria in RTE Foods

#### 3.1.1. Bacterial Load in RTE Foods

Levels of bacterial contamination varied across the analysed food types and processing categories. Artisanally produced (maize-based) foods obviously contained higher TBC and TEC compared to industrially processed (wheat-based) foods. Specifically, *aadun*, a maize-based RTE food from artisanal production, had the highest TBC of 1.33 × 10^7^ and TEC of 6.47 × 10^6^ CFU per gram, but the least counts of 1.69 × 10^6^ and 2.21 × 10^5^ CFU per gram, respectively, were recorded in the wheat-based, industrially produced RTE food, i.e., biscuit (Table 1). The findings in the present study are corroborated by previous reports containing bacterial counts > 10^4^ CFU per gram in RTE foods, which indicate unsafe food contamination levels that could result in severe health risks to consumers of these foods [2,10,34,35]. The disparities in the counts recorded between the two food categories (artisanal vs. industrial) based on their production modes suggest the critical role of maintaining proper hygiene during food handling, especially during food-processing and post-food-processing activities.

#### 3.1.2. Occurrence and Diversity of Bacterial Species in RTE Foods

**Figure 2:**
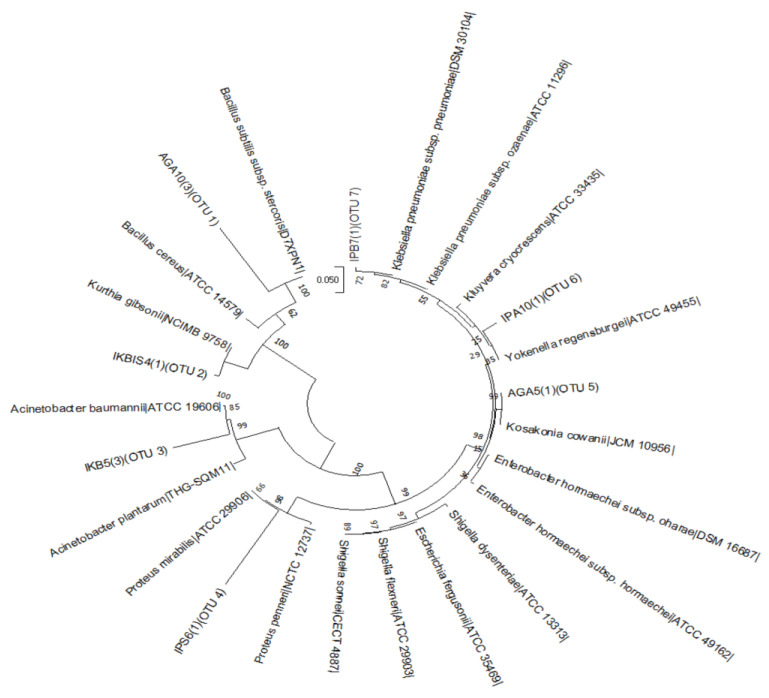
Neighbour-joining phylogenetic tree generated by multiple sequence alignment of the most complete 16S rRNA gene sequences of bacterial isolates obtained from ready-to-eat foods in Lagos (Nigeria) and DNA sequences downloaded from *EZBioCloud* database. The operational taxonomic unit (OTU) clusters of the sequences are indicated, and bootstrap values of 1000 replications are indicated on the tree nodes. Accession numbers of Genbank sequences are in parentheses.

**Figure 3:**
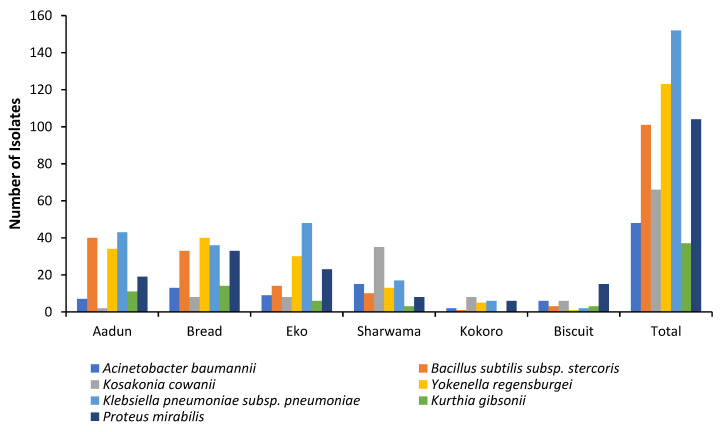
Distribution of 631 bacterial isolates in ready-to-eat foods vended in Lagos, Nigeria.

A total of 631 bacterial isolates were recovered from 92% (183/199) of the RTE foods, and their distributions are *aadun* (n = 156), biscuit (n = 36), bread (n = 177), *eko* (n = 138), shawarma (n = 101), and *kokoro* (n = 28). The NCBI blast search of DNA sequences resulted in ten species (*Acinetobacter baumannii*, *Acinetobacter nosocomialis*, *Bacillus cereus*, *Enterobacter hormaechei*, *Enterobacter* sp., *Escherichia coli*, *Klebsiella pneumoniae*, *Lysinibacillus xylanilyticus*, *Proteus mirabilis,* and *Shigella flexneri*) categorized into eight genera (Table 2). However, based on 16S rRNA gene sequencing similarity at >99%, seven operational taxonomic units (OTUs) were identified (Table 2), and their phylogenetic relatedness is depicted in Figure 2. Taxonomically, the OTUs obtained comprised seven genera: *Klebsiella* (24%), *Yokenella* (20%; consisting of *E. coli* and *S. flexneri*), *Proteus* (17%), *Bacillus* (16%), *Kosakonia* (11%; consisting of *Enterobacter* sp. and *E. hormaechei*), *Acinetobacter* (8%), and *Kurthia* (6%; consisting of *L. xylanilyticus*). The distribution of the isolates, recovered from the RTE foods, at OTU species level is shown in Figure 3. Enteric bacteria, though mostly opportunistic pathogens, widely serve as indicators of faecal contamination of food and water, thereby indicating potential health hazards for consumers [31,36]. Cases of dysentery and diarrhoea have been linked to the consumption of a variety of foods including fruits contaminated with foodborne bacterial species such as *E. coli*, *K. pneumoniae,* and *S. flexneri* [37,38,39,40]. The phylogroups of bacteria recovered in the present study have been widely reported to contaminate RTE foods as a result of poor handling practices and poor personal hygiene of vendors [2,31,36,41,42,43,44,45].

Gram-positive bacteria found in the samples include *Bacillus subtilis* and *Kurthia gibsonii* recovered from all RTE foods investigated except *kokoro*, which did not contain *K. gibsonii* (Figure 3). Both species have been associated with spoilage of RTE foods, especially those containing meat and dairy products [45,46,47,48,49]. Food-spoilage bacteria cause huge economic losses by shelf-life reduction of RTE foods such that the foods become undesirable and unsafe for consumers [50,51]. The Enterobacteriaceae—*A. baumannii*, *K. pneumoniae*, *Kosakonia cowanii*, *P. mirabilis*, and *Yokenella regensburgei*—were present in all the RTE samples (Figure 3). These species have been reported as emerging pathogens, some of which possess antibiotic resistance genes that increase their health-threat status to consumers of RTE foods [52,53,54,55]. For example, a novel strain of *P. mirabilis* was found to be responsible for food poisoning in Shenzhen, China [53]. Previous reports indicated these pathogens in RTE foods vended in Nigeria [31,56,57,58] except *Yokenella*, *Kosakonia,* and *Kurthia,* which are now reported for the first time, to the best of our knowledge, in these foods.

Possible implicating factors that predisposed the RTE foods to bacterial contamination include use of low-quality raw materials, poor processing, and poor handling by vendors, as previously established [12,31,59]. Consequently, adequate enlightenment and education of food vendors and processors on proper food handling and processing as well as good personal hygienic practices will ensure safety of the RTE foods.

#### 3.1.3. Amylolytic and Haemolytic Bacteria in RTE Foods

**Figure 4:**
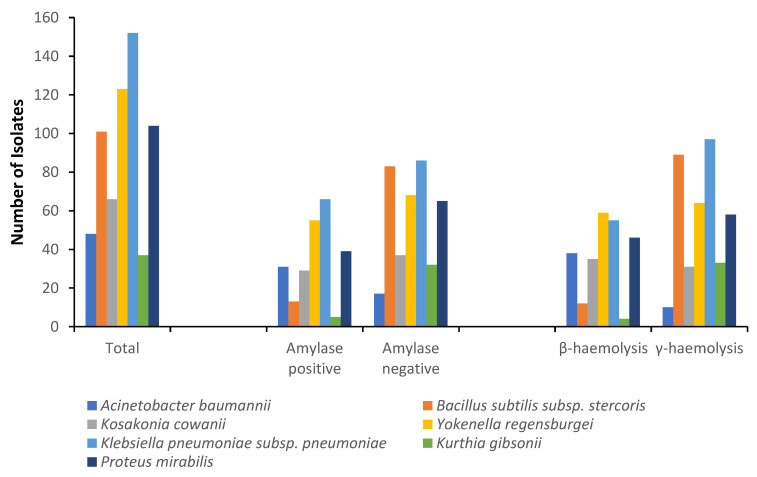
Distribution of haemolysin and amylase production in 631 bacterial isolates from ready-to-eat foods in Lagos, Nigeria.

Amylase is produced by bacteria capable of degrading carbohydrate-containing RTE foods, leading to food spoilage and consequent huge economic losses to vendors [47,60]. In this study, 37.7% (n = 238) of the recovered 631 isolates were positive for amylase production (Figure 4), thus indicating the potential to spoil the RTE foods through starch hydrolysis. Similarly, 39.5% (n = 249) of all the recovered isolates were β-haemolytic, whereas 60.5% (n = 382) were γ-haemolytic (Figure 4). All amylase-producing bacteria were haemolytic, and all recovered bacterial species were represented in the two groups. Gram-positive bacteria (e.g., *Bacillus*) and members of the Enterobacteriaceae (e.g., *K. pneumoniae*) have been reported to exhibit toxigenic properties as a result of their ability to breakdown red blood cells [31,61,62]. Despite showing this property phenotypically, haemolytic assay is a presumptive test and as such must be confirmed by molecular methods. In addition, epidemiological risk-based studies of RTE foods are required to ensure the safety of RTE food consumers, especially children and infants. The underestimated health risks, loss of RTE food quality, and economic losses associated with the presence of haemolytic and spoilage bacteria could result in dire consequences on consumers and vendors of RTE foods, such as severe gastroenteritis and loss of livelihood, respectively.

### 3.2. Biotoxins in the RTE Foods

#### 3.2.1. Overview Data

**Figure 5:**
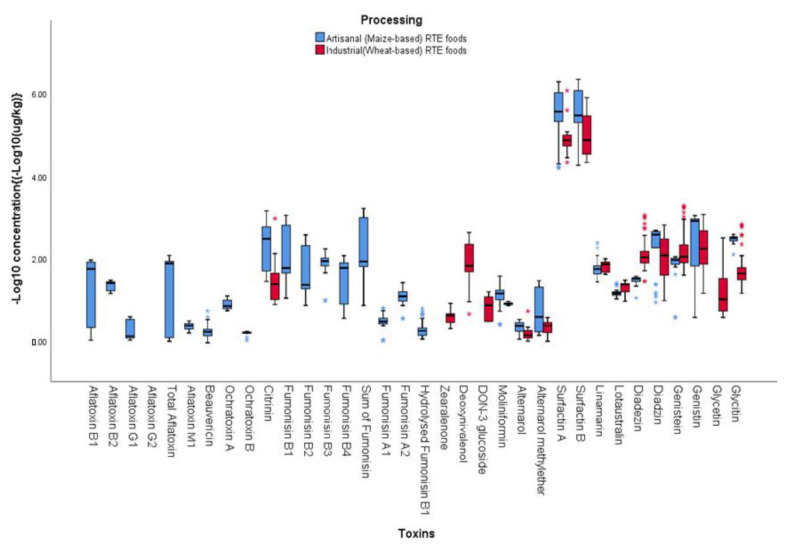
Variation in biotoxins in artisanal and industrial ready-to-eat foods vended in Lagos State, Nigeria. *: outlier data.

A total of 111 metabolites, including 86 fungal metabolites and mycotoxins, 6 phytoestrogenic phenols, 2 plant metabolites/toxins, 2 bacterial metabolites, and 15 metabolites from unspecific sources, were detected in the 199 RTE foods vended in Lagos, Nigeria. Only 107 of the 111 metabolites were quantified; nigragillin, surfactin A, surfactin B, and zearalenone-sulphate were unquantified due to the absence of quantitative standards (Supplementary Table S1). RTE foods produced artisanally (maize-based foods) contained more diverse metabolites compared to the industrially produced (wheat-based) RTE foods (Figure 5, Table 3 and Table 4, and Supplementary Table S2). The disparity in the metabolite profiles of the two food groups (artisanal vs. industrial) may have been due to ingredient type and processing techniques.

#### 3.2.2. Fungal Metabolites and Mycotoxins

Of the 86 fungal metabolites and mycotoxins recovered from the RTE food samples, 69.8% (60/86), 55.8% (48/86), 43% (37/86), 30.2% (26/86), 29.1% (25/86), and 17.4% (15/86) were found in *aadun*, *kokoro*, biscuit, shawarma, bread, and *eko*, respectively (Figure 5, Table 3 and Table 4, and Supplementary Table S2). Only the unspecific diketopiperazine cyclo(L-Pro-L-Tyr) was found in all the 199 investigated food samples (Supplementary Table S2). The spectrum of metabolites including the mycotoxins that were quantified in this study agree with the diversity of metabolites previously reported in various RTE foods [17,18,22,63,64,65,66], although a higher number was found in the present study.

Aflatoxins were found only in the maize-based foods—*aadun* (B-type: 22.9%; G-type: 17.1%), *eko* (B-type: 66.7%), and *kokoro* (B-type: 100%; G-type: 71.4%; AFM_1_: 71.4%) (Table 3). The mean total aflatoxin level was highest in *kokoro* (71.8 µg/kg; range: 2.1–125 µg/kg), occurring in all its samples compared to all the other investigated foods. Fumonisins (B_1_, B_2_, B_3_, B_4_, A_1_, A_2_, and hydrolysed fumonisins) were also found only in *aadun*, *eko,* and *kokoro* (Table 3) and occurred in all samples of *aadun*, with mean total of the B fumonisins being 1080 µg/kg (range: 510–1710 µg/kg). Citrinin was detected in all the RTE food types investigated except for *eko*; the highest incidence (77%) was found in *aadun*, whereas the highest mean concentration (711 µg/kg) was recorded in *kokoro*. Deoxynivalenol was quantified only in the wheat-based RTE foods, specifically in all the biscuit, bread, and 60% of shawarma samples; the highest mean concentration (308 µg/kg; range: 184–422 µg/kg) recorded was in biscuit (Table 4). Zearalenone was detected only in the biscuit, occurring in all its samples at mean level of 4 µg/kg (range: 2.0–8.1 µg/kg) (Table 4). Quantifying aflatoxins, fumonisins, and ochratoxins in only the maize-based RTE foods (*aadun*, *eko,* and *kokoro*) and deoxynivalenol and zearalenone in RTE foods (biscuit, bread, and shawarma) processed from wheat agrees with literature on crop-specific mycotoxin contamination [18,64,67,68,69,70,71,72,73,74,75,76,77,78]. As none of the RTE foods were visibly mouldy, mycotoxin contamination of the foods obviously occurred during preharvest, at harvest, or during grain/raw material storage [79,80]. The presence of the carcinogenic aflatoxins and fumonisins as well as other mycotoxins has a widely documented impact on the overall health of consumers, especially that of children [24,81]. This is the first report of the simultaneous detection of these mycotoxins in *aadun*, biscuit, bread, *eko*, and shawarma in Nigeria. There is therefore a need for continuous surveillance of these foods to understand the extent and patterns of contamination in these RTE foods.

Artisanally processed (maize-based) RTE foods contained significantly (*p* < 0.05) higher mycotoxin levels compared to the industrially processed (wheat-based) RTE foods (Figure 5). Total aflatoxins (mean: 39.0 µg/kg), ochratoxin A and B (means: 7.9 and 1.5 µg/kg, respectively), and total fumonisins (mean: 475 µg/kg) were recorded only in the artisanal RTE foods, while deoxynivalenol (mean: 150 µg/kg) and zearalenone (mean: 4.0 µg/kg) were detected only in industrially processed RTE foods (Figure 5). However, citrinin was detected in RTE foods processed by the two methods, although higher mean values were recorded in artisanal RTE foods (421 µg/kg) compared to industrially processed RTE foods (70.7 µg/kg). (Figure 5). Processing methods have been reported to influence the type of toxins in food [82,83,84]. The detection of aflatoxins (B_1_, B_2_, and G_1_) and fumonisins (B_1_, B_2_, and B_3_) in the artisanal (maize-based) RTE foods is consistent with reports from maize-based snacks from Nigeria [20]. In addition, the presence of aflatoxins, fumonisins, and ochratoxins in only the artisanal RTE foods point to the non-regulation of these RTEs in the local market, as they are not industrially processed [85,86]. The regulation of industries that produce foods make it important for these industries to apply effective quality control in their processes to ensure that the regulated mycotoxins are kept within limits; thus, they may not be found in the foods, as recorded in the present study. In addition, poverty that would cause low or even no access to high-quality grains/crops, poor grain storage, and lack of proper education of artisanal producers of RTE foods on physical sorting of grains are among the factors that may have been responsible for the mycotoxins found in the RTE foods produced artisanally. Despite the hygienic practices deployed in industrial processing of RTE foods, previous reports point to the presence of mycotoxins in these foods, which may also have accounted for the sparing detection of deoxynivalenol and zearalenone in the industrially processed RTE foods in the present study [21,64,67,87]. Although only total aflatoxin is currently regulated in foods in Nigeria at 10 µg/kg, this toxin is not regulated in artisanal foods. In the present study, aflatoxin levels in the artisanally produced RTE exceeded the regulatory limit by at least threefold. Thus, regulatory agencies should ensure that producers of RTE foods adhere to regulatory limits to ensure the safety of consumers. Furthermore, a comprehensive mycotoxin surveillance study of RTE foods available in the informal markets in Nigeria needs to be conducted.

#### 3.2.3. Phytoestrogens

Six phytoestrogens (daidzein, daidzin, genistein, genistin, glycitein, and glycitin) were quantified in the RTE foods investigated in this study. No less than one-half (54%) of the 35 *aadun* samples contained the phytoestrogens; genistin recorded the highest concentration (mean: 615 µg/kg; range: 3.8–1079 µg/kg) (Table 3). Only one sample of *eko* contained 3.8 µg/kg of genistein, while none of the phytoestrogens were detected in *kokoro* (Table 3). For the wheat-based foods (biscuit, bread, and shawarma), the phytoestrogens occurred in all samples except genistein, which was found in 64% of biscuit, and glycitin, which was not detected in bread but was present in 94% of shawarma (Table 4). In biscuit, genistin had the highest mean concentration of 472 µg/kg (range: 400–530 µg/kg), whereas genistein recorded the highest mean concentrations of 146 µg/kg (range: 57.1–309 µg/kg) and 408 µg/kg (range: 39.3–1970 µg/kg) in bread and shawarma, respectively (Table 4). Generally, industrially processed RTE foods contained more phytoestrogens compared to the artisanal RTE foods (Figure 5). For example, mean daidzein, genistein, and glycitein concentrations were 161 µg/kg vs. 29.7 µg/kg, 221 µg/kg vs. 77.3 µg/kg, and 31.9 µg/kg vs. not detected in industrial and artisanal RTE foods, respectively (Figure 5). Phytoestrogens (e.g., daidzein, daidzin, genistein, genistin, and glycitin) are predominantly found in soybean but also found in other legumes and cereals usually used in RTE foods production [88]. However, this is the first report of the six phytoestrogens in RTE foods in Nigeria. The phytoestrogens are known to have beneficial effects in cancer treatment [89,90]; however, they also possess non-beneficial effects as endocrine disruptors and possess epigenetic effects that could cause adverse health risks to consumers [91,92,93]. In view of the controversial nature of the roles of these phytoestrogens, further studies on the individual toxicological impacts of these compounds and their effects when combined with other classes of biotoxins reported in this study is urgent.

#### 3.2.4. Plant Toxins

Linamarin and lotaustralin, two phytotoxins, were detected only in *aadun*, biscuit, and bread (Table 3 and Table 4). Linamarin had higher mean levels (53.2, 71.0, and 79.9 µg/kg) in the foods compared to lotaustralin (Table 3 and Table 4). Both phytotoxins were detected in both industrial and artisanal RTE foods (Figure 5). Linamarin and lotaustralin have been reported to be found at 3–4 orders of magnitude higher in cassava flour [94], an ingredient usually added in trace amounts to improve the mouthfeel of RTE foods such as *aadun*, biscuits, and bread. Although the concentrations of the compounds in the RTE foods investigated in the present study were very low, these plant toxins have been associated with acute cyanide poisoning, which along with chronic exposure of the two phytotoxins potentially results in negative health consequences, especially in young children [94,95].

#### 3.2.5. Bacterial Metabolites

Two bacterial metabolites (surfactin A and surfactin B) contaminated all RTE food types except biscuit (Table 3 and Table 4). These metabolites were detected the most in *aadun* compared to other food types. Despite reports of bacterial metabolites in the ingredients as well as final products [3,17], this is the first report of surfactin A and B in RTE foods. Adetunji et al. [96] also reported low concentrations of bacterial metabolites in stored maize, which is a critical ingredient in artisanal RTE foods. Of note is the fact that due to a limited target of the LC-MS/MS method towards bacterial metabolites, there is a weak link between the bacteria isolates and the bacterial metabolites recovered from this study. Nevertheless, the roles of the detected compounds and their combinations with mycotoxins need further investigation. In addition, there is a need to further study the routes of contamination of these metabolites in RTE food.

## 4. Conclusions

RTE foods in this study were shown to contain diverse food contaminants ranging from pathogenic bacteria, fungal toxins, phytoestrogens, and plant toxins to bacterial metabolites. Bacterial contaminants were found in all the food types, but lower distribution was found in visibly dried foods (e.g., *kokoro*) with industrial packaging (e.g., biscuit) compared to those with higher moisture content and packaged by personnel who may have poor hygiene. The study further showed that artisanal (maize-based) RTE foods contained more biotoxins compared to industrially processed (wheat-based) RTE foods. Hence, the consumption of artisanal RTE foods could pose risks of foodborne illnesses and mycotoxicosis to consumers, especially children who heavily depend on RTE foods such as *aadun*, biscuits, *eko,* and *kokoro*. The findings of this study suggest that routine surveillance, especially of artisanal RTE foods available in informal markets, be conducted in Lagos State, the most populous state in and a major industrial hub of the nation and Africa, and education to food processors and handlers should be given to enhance food quality and safeguard consumer health. There is also a need to ensure that high-quality raw materials are sourced to ensure safe products for consumers towards actualizing goal 3 (good health and wellbeing) of the United Nations sustainable development goals in the country.

## Figures and Tables

**Table 1:** Total bacterial count (TBC) and total enterobacterial count (TEC) in vended ready-to eat foods in Lagos, Nigeria.

RTE Food Processing Category	RTE Food Type	TBC (CFU per g)			TEC (CFU per g)		
		Range	Mean	SD	Range	Mean	SD
Artisanal	*Aadun* (n = 35)	1.0 × 10^7^–1.7 × 10^7^	1.33 × 10^7^	1.71 × 10^6^	3.0 × 10^6^–1.0 × 10^7^	6.47 × 10^6^	1.71 × 10^6^
	*Eko* (n = 30)	3.0 × 10^5^–2.0 × 10^7^	1.00 × 10^7^	2.24 × 10^6^	1.0 × 10^5^–1.0 × 10^7^	4.78 × 10^6^	2.38 × 10^6^
	*Kokoro* (n = 35)	4.0 × 10^5^–1.0 × 10^7^	3.58 × 10^6^	5.53 × 10^6^	1.0 × 10^5^–3.0 × 10^6^	2.91 × 10^5^	2.81 × 10^6^
Industrial	Biscuit (n = 34)	1.0 × 10^5^–1.0 × 10^7^	1.69 × 10^6^	2.25 × 10^6^	1.0 × 10^5^–2.0 × 10^6^	2.21 × 10^5^	1.72 × 10^6^
	Bread (n = 30)	6.0 × 10^6^–2.0 × 10^7^	1.23 × 10^7^	2.09 × 10^6^	1.0 × 10^6^–1.0 × 10^7^	5.89 × 10^6^	3.72 × 10^5^
	Shawarma (n = 35)	2.0 × 10^6^–1.0 × 10^7^	7.51 × 10^6^	2.83 × 10^6^	2.0 × 10^5^–5.0 × 10^6^	2.27 × 10^6^	6.99 × 10^5^

SD, standard deviation.

**Table 2:** Phylogenetic similarities of 16S rRNA gene operational taxonomic units (OTU) of isolates from ready-to-eat foods vended in Lagos, Nigeria.

OTU Cluster Number	Lab ID of OTU	Isolates per OTU Cluster	*EzBioCloud* Database		Accession Number of Sequences	Species in OTU Based on NCBI Blast
			Closest Match	Similarity (%)		
OTU1	AGA10(3)	101	*Bacillus subtilis* subsp. *stercoris*	99.56	MW448726	*Bacillus cereus*
OTU2	IKBIS4(1)	37	*Kurthia gibsonii*	99.76	MW448724	*Lysinibacillus xylanilyticus*
OTU3	IKB5(3)	48	*Acinetobacter baumannii*	99.76	MW448712	*Acinetobacter baumannii* ×2, *A. nosocomialis*
OTU4	IPS6(1)	104	*Proteus mirabilis*	100	MW448727	*Proteus mirabilis* ×2
OTU5	AGA5(1)	66	*Kosakonia cowannii*	100	MW448732	*Enterobacter* sp., *Enterobacter hormaechei* ×2,
OTU6	IPA10(1)	123	*Yokenella regensburgei*	99.4	MW448714	*Escherichia coli* ×2, *Shigella flexneri* ×2
OTU7	IPB7(1)	152	*Klebsiella pneumoniae* subsp. *pneumoniae*	99.76	MW448717	*Klebsiella pneumoniae* ×3

**Table 3:** Regulated mycotoxins, phytoestrogens, phytotoxins, and bacterial metabolites in maize-based ready-to-eat foods vended in Lagos, Nigeria.

Biotoxins	*Aadun* (n = 35)			*Eko* (n = 30)			*Kokoro* (n = 35)		
	%p	Concentration (µg/kg)		%p	Concentration (µg/kg)		%p	Concentration (µg/kg)	
		Range	Mean		Range	Mean		Range	Mean
*Regulated mycotoxins*									
Aflatoxin B_1_	22.9	0.4–1.1	0.6	66.7	0.4–1.9	0.7	100	1.8–90.6	52.2
Aflatoxin B_2_	0.0	<LOD	<LOD	0.0	<LOD	<LOD	100	0.3–29.8	16.2
Aflatoxin G_1_	17.1	0.3–0.7	0.3	0.0	<LOD	<LOD	71.4	0.8–3.9	2.1
Aflatoxin G_2_	0.0	<LOD	<LOD	0.0	<LOD	<LOD	22.9	0.9	0.9
Total aflatoxin	28.6	0.3–1.8	0.7	66.7	0.4–1.9	0.7	100	2.1–125	71.8
Aflatoxin M_1_	0.0	<LOD	<LOD	0.0	<LOD	<LOD	71.4	1.6–3.1	2.4
Ochratoxin A	0.0	<LOD	<LOD	0.0	<LOD	<LOD	71.4	5.4–12.3	7.9
Ochratoxin B	0.0	<LOD	<LOD	0.0	<LOD	<LOD	28.6	1.1–1.7	1.5
Citrinin	77.1	27.6–577	153	0.0	<LOD	<LOD	71.4	278–1412	711
Fumonisin B_1_	100	315–1100	685	46.7	10.9–31.9	18.7	100	34.6–62.9	51.1
Fumonisin B_2_	100	111–369	222	36.7	7.3–14.4	11.2	100	15.9–25.8	20.6
Fumonisin B_3_	100	44.7–170	92.3	0.0	<LOD	<LOD	11.4	9.6	9.6
Fumonisin B_4_	100	22.2–117	67.7	0.0	<LOD	<LOD	42.9	3.5–8.9	5.0
Fumonisin A_1_	100	1.1–6.3	3.0	0.0	<LOD	<LOD	0.0	<LOD	<LOD
Fumonisin A_2_	91.4	3.5–26.0	12.2	0.0	<LOD	<LOD	0.0	<LOD	<LOD
Hydrolysed FB_1_	100	0.3–6.1	2.4	0.0	<LOD	<LOD	100	1.0–2.0	1.5
Total fumonisin	100	510–1707	1081	53.3	7.3–44.4	24.0	100	57.6–97.0	76.4
Zearalenone	0.0	<LOD	<LOD	0.0	<LOD	<LOD	0.0	<LOD	<LOD
Deoxynivalenol	0.0	<LOD	<LOD	0.0	<LOD	<LOD	0.0	<LOD	<LOD
*Plant toxins*									
Linamarin	82.9	26.9–239	71.0	0.0	<LOD	<LOD	0.0	<LOD	<LOD
Lotaustralin	71.4	10.4–25.4	15.0	0.0	<LOD	<LOD	0.0	<LOD	<LOD
*Phytoestrogens*									
Daidzein	54.3	11.1–37.0	29.7	0.0	<LOD	<LOD	0.0	<LOD	<LOD
Daidzin	71.4	8.6-473	306	0.0	<LOD	<LOD	0.0	<LOD	<LOD
Genistein	60.0	3.8–110	80.8	3.3	3.8	3.8	0.0	<LOD	<LOD
Genistin	74.3	3.8–1079	615	0.0	<LOD	<LOD	0.0	<LOD	<LOD
Glycitein	0.0	<LOD	<LOD	0.0	<LOD	<LOD	0.0	<LOD	<LOD
Glycitin	54.3	124–380	291	0.0	<LOD	<LOD	0.0	<LOD	<LOD
*Bacterial metabolites*									
Surfactin A	94.3	110,640–1,946,400	1,061,765	43.3	16,024-151,680	45,774	71.4	213,520–448,640	331,533
Surfactin B	97.1	157,920–2,239,200	1,105,019	26.7	18,672–170,800	72,753	71.4	185,760–320,240	253,411

**Table 4:** Regulated mycotoxins, phytoestrogens, phytotoxins, and bacterial metabolites in wheat-based ready-to-eat foods vended in Lagos, Nigeria.

Biotoxins	Biscuit (n = 34)			Bread (n = 30)			Shawarma (n = 35)		
	%p	Concentration (µg/kg)		%p	Concentration (µg/kg)		%p	Concentration (µg/kg)	
		Range	Mean		Range	Mean		Range	Mean
*Regulated mycotoxins*									
Aflatoxin B_1_	0.0	<LOD	<LOD	0.0	<LOD	<LOD	0.0	<LOD	<LOD
Aflatoxin B_2_	0.0	<LOD	<LOD	0.0	<LOD	<LOD	0.0	<LOD	<LOD
Aflatoxin G_1_	0.0	<LOD	<LOD	0.0	<LOD	<LOD	0.0	<LOD	<LOD
Aflatoxin G_2_	0.0	<LOD	<LOD	0.0	<LOD	<LOD	0.0	<LOD	<LOD
Total aflatoxin	0.0	<LOD	<LOD	0.0	<LOD	<LOD	0.0	<LOD	<LOD
Aflatoxin M_1_	0.0	<LOD	<LOD	0.0	<LOD	<LOD	0.0	<LOD	<LOD
Ochratoxin A	0.0	<LOD	<LOD	0.0	<LOD	<LOD	0.0	<LOD	<LOD
Ochratoxin B	0.0	<LOD	<LOD	0.0	<LOD	<LOD	0.0	<LOD	<LOD
Citrinin	2.9	10.9	10.9	23.3	8.1–14.3	10.2	40.0	7.6–918	105
Fumonisin B_1_	0.0	<LOD	<LOD	0.0	<LOD	<LOD	0.0	<LOD	<LOD
Fumonisin B_2_	0.0	<LOD	<LOD	0.0	<LOD	<LOD	0.0	<LOD	<LOD
Fumonisin B_3_	0.0	<LOD	<LOD	0.0	<LOD	<LOD	0.0	<LOD	<LOD
Fumonisin B_4_	0.0	<LOD	<LOD	0.0	<LOD	<LOD	0.0	<LOD	<LOD
Fumonisin A_1_	0.0	<LOD	<LOD	0.0	<LOD	<LOD	0.0	<LOD	<LOD
Fumonisin A_2_	0.0	<LOD	<LOD	0.0	<LOD	<LOD	0.0	<LOD	<LOD
Hydrolysed FB_1_	0.0	<LOD	<LOD	0.0	<LOD	<LOD	0.0	<LOD	<LOD
Total fumonisin	0.0	<LOD	<LOD	0.0	<LOD	<LOD	0.0	<LOD	<LOD
Zearalenone	100	2.0–8.1	4.0	0.0	<LOD	<LOD	0.0	<LOD	<LOD
Deoxynivalenol	100	184–422	308	100	43.9–87.1	59.8	60.0	4.5–56.9	23.6
*Plant toxins*									
Linamarin	29.4	41.2–92.0	53.2	33.3	71.5–97.6	79.9	0.0	<LOD	<LOD
Lotaustralin	0.0	<LOD	<LOD	90.0	9.2–29.7	20.7	0.0	<LOD	<LOD
*Phytoestrogens*									
Daidzein	100	75.2–123	98.6	100	50.0–276	130	100	27.7–1100	250
Daidzin	100	267–341	299	100	14.0–145	77.1	100	9.4–641	118
Genistein	64.1	116–93.4	93.4	100	57.1–309	146	100	39.3–1970	408
Genistin	100	400–530	472	100	18.8–201	108	100	14.3–1145	212
Glycitein	100	3.8–13.1	8.1	100	3.8–34.4	13.4	100	3.8–311	71.0
Glycitin	100	20.5–50.7	34.7	0.0	<LOD	<LOD	94.3	14.3–665	141
*Bacterial metabolites*									
Surfactin A	0.0	<LOD	<LOD	20.0	21,872–1,186,400	309,260	31.4	28,368–116,320	699,19
Surfactin B	0.0	<LOD	<LOD	16.7	21,816-800,800	244,734	2.9	75,152–75,152	75,152

## Data Availability

Data are available on request from authors.

## Supplementary Materials

The following supporting information can be downloaded at: https://www.mdpi.com/article/10.3390/foods12061224/s1, Table S1: LC-MS/MS limits of detection (LOD) and limit of quantification (LOQ) for 111 metabolites in ready-to-eat foods; Table S2: Occurrence levels of non-regulated metabolites in ready-to-eat foods vended in Lagos, Nigeria.
