# Supplementary material for: Bacteriological Quality and Biotoxin Profile of Ready-to-Eat Foods Vended in Lagos, Nigeria
Source: Foods. 2023 Mar 13;12(6):1224. doi: 10.3390/foods12061224 (PMC10048420; doi:10.3390/foods12061224)
Supplement: Supplementary file 1 [file foods-12-01224-s001.zip › foods-2239490-supplementary.pdf]

Bacteriological quality and biotoxin profile of ready-to-eat foods vended in Lagos, Nigeria

Oluwadamilola M. Makinde [1,2,3], Michael Sulyok [4], Rasheed A. Adeleke [2], Rudolf Krska [4,5] and Chibundu N. Ezekiel [1,4,*]

[1] Department of Microbiology, Babcock University, Ilishan Remo 121103, Ogun State, Nigeria
[2] Unit for Environmental Sciences and Management, North-West University (Potchefstroom Campus), Potchefstroom 2531, South Africa
[3] Department of Biological Sciences and Biotechnology, Caleb University, Lagos 106102, Nigeria
[4] Department of Agrobiotechnology IFA-Tulln, Institute of Bioanalytics and Agro-Metabolomics, University of Natural Resources and Life Sciences, Vienna, Konrad-Lorenz-Strasse 20, 3430 Tulln an der Donau, Austria
[5] Institute for Global Food Security, School of Biological Sciences, Queen's University Belfast, University Road, 19 Chlorine Gardens, Belfast BT9 5DL, Northern Ireland, UK
* Correspondence: chaugez@gmail.com or chibundu.ezekiel@boku.ac.at

Supplementary Tables

Table S1. LC-MS/MS limits of detection (LOD) and limit of quantification (LOQ) for 111 metabolites in ready-to-eat foods.

Metabolite	LOD (µg/kg)	LOQ (µg/kg)	Metabolite	LOD (µg/kg)	LOQ (µg/kg)
15-Hydroxyculmorin	6.2	20.8	Endocrocin	15.4	51.2
3-Nitropropionic acid	0.7	2.5	Enniatin A	0.0	0.0
5-Hydroxyculmorin	25.0	75.0	Enniatin A1	0.1	0.2
7-Hydroxypestalotin	0.5	1.6	Enniatin B	0.0	0.0
Aflatoxin B_1	0.2	0.7	Enniatin B1	0.0	0.1
Aflatoxin B_2	0.1	0.2	Enniatin B2	0.2	0.6
Aflatoxin G_1	0.2	0.5	Epiequisetin	0.1	0.3
Aflatoxin G_2	0.5	1.7	Equisetin	0.1	0.3
Aflatoxin M_1	0.1	0.4	Fellutanine A	1.0	3.3
Agroclavine	0.1	0.3	Festuclavine	0.0	0.1
Alternariol	0.1	0.3	Flavoglaucin	0.0	0.1
Alternariolmethylether	0.2	0.5	Fumigaclavine C	0.8	2.8
Altersetin	1.1	3.6	Fumonisin A_1	0.8	2.1
Andrastin A	0.2	0.8	Fumonisin A_2	2.1	7.1
Ascochlorin	0.1	0.2	Fumonisin B_1	2.4	8.0
Asperfuran	6.4	21.5	Fumonisin B_2	2.1	7.1
Asperglaucide	0.1	0.2	Fumonisin B_3	5.8	19.2
Asperphenamate	0.4	1.3	Fumonisin B_4	2.1	7.1
Aspochracin	1.0	3.0	Genistein	2.5	7.5
Aurofusarin	1.1	3.7	Genistin	2.5	7.5
Averufin	0.0	0.1	Gliotoxin	1.2	4.0
Beauvericin	0.0	0.1	Glycitein	2.5	7.5
Berkedrimane B	0.4	1.2	Glycitin	2.5	7.5
Bikaverin	0.5	1.5	Hydrolysed FB_1	0.2	0.7
Bis(methylthio)gliotoxin	1.0	3.5	Ilicicolin A	0.0	0.1
Brevianamid F	1.0	3.3	Ilicicolin B	0.6	1.9
Chanoclavin	0.2	0.6	Ilicicolin E	0.0	0.1
Chlorocitreorosein	0.8	2.7	Ilicolin C	0.2	0.8
Chrysogin	0.6	1.9	Iso-Rhodoptilometrin	0.0	0.1
Chrysophanol	4.1	13.8	Kojic acid	20.4	67.8
Citreorosein	0.7	2.5	Linamarin	2.3	7.8
Citrinin	0.7	2.5	LL-Z 1272e	0.0	0.1
Culmorin	1.6	5.5	Lotaustralin	1.3	4.4
Curvularin	0.6	2.0	Macrosporin	0.1	0.4
cyclo(L-Pro-L-Tyr)	1.2	3.9	Methylsulochrin	0.0	0.1
cyclo(L-Pro-L-Val)	1.2	3.9	Moniliformin	1.5	5.1
Daidzein	2.5	7.5	Monocerin	0.1	0.2
Daidzin	2.5	7.5	N-Benzoyl-Phenylalanine	0.2	0.5
Deoxynivalenol	3.0	9.0	Neoechinulin A	1.4	4.5
Destruxin B	0.3	1.1	Norsolorinic acid	0.1	0.2
Diacetoxyscirpenol	0.2	0.5	Ochratoxin A	0.5	1.5
Dichlordiaportin	0.6	2.1	Ochratoxin B	0.1	0.4
Dihydrocitrinone	1.1	3.8	*O*-Methylsterigmatocystin	0.1	0.2
DON-3-glucoside	2.0	6.0	Pestalotin	1.2	4.0
Elymoclavine	0.3	0.9	Phenopyrrozin	0.4	1.3
Emodin	0.1	0.4	Pinselin	0.7	2.2

Table S1. (Continued).

Metabolite	LOD (µg/kg)	LOQ (µg/kg)	Metabolite	LOD (µg/kg)	LOQ (µg/kg)
Purpactin A	0.4	1.2	Siccanol	25.0	75.0
Questiomycin A	0.8	2.7	Sterigmatocystin	0.1	0.3
Quinolactacin A	0.0	0.0	Tentoxin	0.1	0.4
Quinolactacin B	0.0	0.0	Tryptophol	3.9	12.9
Radicicol	0.2	0.6	Versicolorin C	0.1	0.5
Rugulovasine A	7.6	25.5	W493	1.0	3.0
Sclerotin A	1.0	3.0	Zearalenone	0.2	0.6
Secalonic acid D	0.9	2.9			

Table S2. Occurrence levels of non-regulated metabolites in ready-to-eat foods vended in Lagos, Nigeria.

Mycotoxin	Artisanally-processed (n = 100)				Industrially-processed (n=99)			
	%p	Concentration (µg/kg)			%p	Concentration (µg/kg)		
		Range	Mean	Median		Range	Mean	Median
15-Hydroxyculmorin	0.0	<LOD	<LOD	<LOD	91.9	10.4-404	118	47.7
3-Nitropropionic acid	73.0	1.2-23.6	9.8	5.0	35.4	5.0-12.8	8.3	7.3
5-Hydroxyculmorin	0.0	<LOD	<LOD	<LOD	34.3	37.5-434	249	270
7-Hydroxypestalotin	69.0	0.8-8.4	3.3	3.2	0.0	<LOD	<LOD	<LOD
Agroclavine	1.0	0.5	0.5	0.5	0.0	<LOD	<LOD	<LOD
Altersetin	2.0	1.8-11.8	6.8	6.8	59.6	1.8-21.3	9.8	12.6
Andrastin A	21.0	6.4-23.2	14.4	12.1	0.0	<LOD	<LOD	<LOD
Ascochlorin	35.0	3.6-18.0	12.6	12.8	7.1	0.4-0.6	0.5	0.5
Asperfuran	25.0	116-244	167	163	0.0	<LOD	<LOD	<LOD
Asperglaucide	94.0	0.1-1413	299	14.0	100	0.8-135	7.9	4.0
Asperphenamate	88.0	0.7-42.6	10.8	2.7	100	0.7-36.1	6.8	4.3
Aspochracin	25.0	24.6-45.1	35.4	39.0	0.0	<LOD	<LOD	<LOD
Aurofusarin	1.0	5.5	5.5	5.5	34.3	80.5-162	127	132
Averufin	60.0	0.2-1.2	0.7	0.6	1.0	0.5	0.5	0.5
Berkedrimane B	5.0	4.5-7.5	6.0	5.9	0.0	<LOD	<LOD	<LOD
Bikaverin	80.0	2.9-33.2	19.2	19.8	0.0	<LOD	<LOD	<LOD
Bis(methylthio)gliotoxin	35.0	1.7-41.2	21.6	22.7	0.0	<LOD	<LOD	<LOD
Brevianamid F	81.0	1.7-23.5	8.5	5.2	100	3.9-102	24.4	16.3
Chanoclavin	0.0	<LOD	<LOD	<LOD	21.2	0.3-0.9	0.5	0.3
Chlorocitreorosein	20.0	1.4-7.9	5.4	5.9	0.0	<LOD	<LOD	<LOD
Chrysogin	0.0	<LOD	<LOD	<LOD	34.3	6.6-14.7	10.9	12.5
Chrysophanol	28.0	21.2-104	62.0	69.9	0.0	<LOD	<LOD	<LOD
Citreorosein	60.0	3.4-29.1	13.0	12.9	1.0	12.4	12.4	12.4
Culmorin	0.0	<LOD	<LOD	<LOD	88.9	16.5-337	122	96.5
Curvularin	10.0	1.0-4.4	3.2	3.5	0.0	<LOD	<LOD	<LOD
cyclo(L-Pro-L-Tyr)	100	1.9-347	76.5	26.8	100	29.7-377	140	126
cyclo(L-Pro-L-Val)	88.0	1.9-261	82.4	47.6	100	7.6-394	121	56.5
Destruxin B	15.0	0.6-1.9	1.0	0.6	0.0	<LOD	<LOD	<LOD
Diacetoxyscirpenol	8.0	0.5-1.1	0.7	0.6	0.0	<LOD	<LOD	<LOD
Dichlordiaportin	35.0	16.9-70.1	37.9	32.1	32.3	4.4-92.6	35.7	15.4
Elymoclavine	1.0	0.4	0.4	0.4	0.0	<LOD	<LOD	<LOD
Emodin	80.0	0.2-59.5	21.8	16.7	68.7	0.2-12.9	0.8	0.5
Endocrocin	21.0	25.6	25.6	25.6	0.0	<LOD	<LOD	<LOD
Enniatin A	0.0	<LOD	<LOD	<LOD	83.8	0.02-0.24	0.1	0.1
Enniatin A1	0.0	<LOD	<LOD	<LOD	99.0	0.1-4.4	1.6	1.6
Enniatin B	0.0	<LOD	<LOD	<LOD	100	0.1-7.6	3.0	2.5
Enniatin B1	0.0	<LOD	<LOD	<LOD	100	0.1-10.8	4.4	3.9
Enniatin B2	0.0	<LOD	<LOD	<LOD	25.3	0.2-0.4	0.3	0.3
Epiequisetin	70.0	1.6-12.6	4.1	3.7	13.1	0.2-3.1	0.5	0.2
Equisetin	70.0	1.5-23.6	4.6	3.2	13.1	0.2-3.0	0.6	0.3
Fellutanine A	0.0	<LOD	<LOD	<LOD	60.6	1.7-26.3	11.7	11.1
Festuclavine	12.0	0.04-0.88	0.6	0.7	0.0	<LOD	<LOD	<LOD
Flavoglaucin	93.0	1.2-318	43.1	8.8	99.0	4.9-71.6	25.1	24.0
Fumigaclavine C	33.0	1.4-11.3	5.7	5.4	0.0	<LOD	<LOD	<LOD

Table S2. Continued.

Mycotoxin	Artisanally-processed (n = 100)				Industrially-processed (n=99)			
	%p	Concentration (µg/kg)			%p	Concentration (µg/kg)		
		Range	Mean	Median		Range	Mean	Median
Gliotoxin	23.0	2.0-9.6	6.5	7.3	0.0	<LOD	<LOD	<LOD
Ilicicolin A	55.0	0.2-12.8	6.1	7.3	15.1	0.1-0.3	0.2	0.2
Ilicicolin B	35.0	4.8-29.2	15.6	15.4	9.1	0.6-1.0	0.8	0.8
Ilicicolin E	36.0	0.1-0.5	0.3	0.3	0.0	<LOD	<LOD	<LOD
Ilicolin C	15.0	2.7-12.5	7.2	7.3	0.0	<LOD	<LOD	<LOD
Iso-Rhodoptilometrin	60.0	3.0-16.9	10.9	10.9	1.0	1.2	1.2	1.2
Kojic acid	90.0	33.9-418	194	138	99.0	33.9-385	151	130
LL-Z 1272e	35.0	0.1-0.5	0.36	0.36	0.0	<LOD	<LOD	<LOD
Macrosporin	22.0	0.2-0.7	0.3	0.2	45.5	0.2	0.2	0.2
Methylsulochrin	34.0	0.6-6.9	2.4	2.4	1.0	0.2	0.2	0.2
Monocerin	81.0	0.1-8.7	2.4	1.7	26.3	0.1	0.1	0.1
N-Benzoyl-Phenylalanine	67.0	0.3-36.7	9.3	2.1	83.8	0.3-30.8	5.1	2.0
Neoechinulin A	45.0	2.3-144	40.8	14.9	52.5	2.3-12.9	7.4	8.5
Norsolorinic acid	26.0	0.5-1.6	0.8	0.8	0.0	<LOD	<LOD	<LOD
O-Methylsterigmatocystin	26.0	0.3-0.6	0.5	0.5	0.0	<LOD	<LOD	<LOD
Pestalotin	73.0	2.0-13.7	5.2	2.0	0.0	<LOD	<LOD	<LOD
Phenopyrrozin	19.0	0.6-2.7	1.7	1.6	0.0	<LOD	<LOD	<LOD
Pinselin	58.0	1.1-15.1	8.6	8.9	1.0	10.0	10.0	10.0
Purpactin A	35.0	2.0-9.3	5.5	5.9	0.0	<LOD	<LOD	<LOD
Questiomycin A	90.0	1.4-40.6	13.8	10.3	0.0	<LOD	<LOD	<LOD
Quinolactacin A	99.0	0.1-70.8	14.7	0.4	21.2	0.02-0.09	0.1	0.1
Quinolactacin B	73.0	0.02-11.0	2.8	0.1	0.0	<LOD	<LOD	<LOD
Radicicol	34.0	2.3-27.4	8.5	5.6	2.0	0.3-0.7	0.5	0.5
Rugulovasine A	33.0	12.8-51.1	22.9	12.8	0.0	<LOD	<LOD	<LOD
Sclerotin A	56.0	1.8-141	50.8	14.0	1.0	81.6	81.6	81.6
Secalonic acid D	0.0	<LOD	<LOD	<LOD	58.6	6.2-51.1	21.5	16.9
Siccanol	21.0	37.5-1023	256	128	0.0	<LOD	<LOD	<LOD
Sterigmatocystin	35.0	0.1-0.8	0.4	0.4	6.1	0.1	0.1	0.1
Tentoxin	0.0	<LOD	<LOD	<LOD	34.3	0.2-0.8	0.5	0.6
Tryptophol	93.0	6.4-1199	60.7	56.8	100	41.9-15769	1529	190
Versicolorin C	57.0	0.3-5.7	2.2	0.7	1.0	3.5	3.5	3.5
W493	0.0	<LOD	<LOD	<LOD	26.3	1.5-17.6	2.7	1.5
